# Supplementary material for: Alarm-assisted urotherapy for daytime urinary incontinence in children: A meta-analysis
Source: PLoS One. 2023 Feb 3;18(2):e0275958. doi: 10.1371/journal.pone.0275958 (PMC9897563; doi:10.1371/journal.pone.0275958)
Supplement: S1 Appendix — (DOCX) [file pone.0275958.s002.docx]

Supplementary 1.

Systematic review

“Alarm-assisted urotherapy for daytime urinary incontinence in children. A meta-analysis”

**Search strategies systematic review**

Search terms MEDLINE

((((("Clinical Alarms"[Mesh] OR "Biosensing Techniques"[Mesh] OR Alarm*[tiab] OR Timed voiding [tiab] OR Timer*[tiab] OR bladder sensor*[tiab] OR bladder notification*[tiab] OR Urotherap* [tiab] OR bladder re-education [tiab] OR bladder rehabilitation [tiab] OR bladder training [tiab] OR voiding school* [tiab] OR "voiding class"[tiab] OR "voiding classes"[tiab])))) **AND ((**Infan* OR newborn* OR new-born* OR perinat* OR neonat* OR baby OR baby* OR babies OR toddler* OR minors OR minors* OR boy OR boys OR boyfriend OR boyhood OR girl* OR kid OR kids OR child OR child* OR children* OR schoolchild* OR schoolchild OR school child[tiab] OR school child*[tiab] OR adolescen* OR juvenil* OR youth* OR teen* OR under*age* OR pubescen* OR pediatrics[mh] OR pediatric* OR paediatric* OR peadiatric* OR school[tiab] OR school*[tiab] OR prematur* OR preterm*))) **AND** ("urinary incontinence"[MeSH ] OR urinary incontinen*[Tiab] OR Urinary urge incontinen*[tiab] OR Daytime incontinen*[tiab] OR “wetting”[tiab] OR “Diurnal enuresis"[MeSH] OR “Diurnal enuresis”[Tiab] OR "Lower Urinary Tract Symptoms"[Mesh:NoExp] OR "Urinary Bladder, Overactive"[Mesh] OR lower urinary tract symptom*[Tiab] OR overactive bladder[tiab] OR overactive urinary bladder[tiab] OR Overactive Detrusor[tiab] OR urine incontinen*[tiab] OR bladder incontinen*[tiab] OR urinary leakage [tiab] OR urine leakage[tiab] OR bladder overactivity [tiab] OR detrusor overactivity [tiab] OR micturition disorder*[tiab] OR micturition disturbance*[tiab] OR lower urinary tract dysfunction [tiab])

Search strategies EMBASE

(micturition disorder/ or lower urinary tract symptom/ OR diurnal enuresis/ OR urine incontinence/ or urge incontinence/ OR overactive bladder/ OR bladder training/ OR daytime incontinence.dj. OR (bladder overactivity OR detrusor overactivity OR micturition disorder* OR micturition disturbance* OR lower urinary tract dysfunction OR Urine incontinen* OR Bladder incontinen* OR Urinary leakage OR Urine leakage OR urinary incontinen* OR Urinary urge incontinen* OR Daytime incontinen* OR wetting OR Diurnal enuresis OR lower urinary tract symptom* OR overactive bladder OR overactive urinary bladder OR Overactive Detrusor).ti,ab,kw.) AND (Infan* OR newborn* OR new-born* OR perinat* OR neonat* OR baby OR baby* OR babies OR toddler* OR minors OR minors* OR boy OR boys OR boyfriend OR boyhood OR girl* OR kid OR kids OR child OR child* OR children* OR schoolchild* OR schoolchild OR school child OR school child* OR adolescen* OR juvenil* OR youth* OR teen* OR under*age* OR pubescen* OR pediatrics OR pediatric* OR paediatric* OR peadiatric* OR school OR school* OR prematur* OR preterm*).mp. AND Sensor/ or biosensor/ or alarm monitor/ or bladder training/ or (Alarm* or Timed voiding or Timer* or bladder sensor* or bladder notification*).ti,ab,kw. or (urotherap* or bladder re-education or bladder rehabilitation or bladder training or voiding school* or voiding class*).ti,ab,kw.

Search items PsychINFO

(((urinary incontinence/ or urinary function disorders/) or (bladder overactivity OR detrusor overactivity OR micturition disorder* OR micturition disturbance* OR lower urinary tract dysfunction OR Urine incontinen* OR Bladder incontinen* OR Urinary leakage OR Urine leakage OR urinary incontinen* OR Urinary urge incontinen* OR Daytime incontinen* OR wetting OR Diurnal enuresis OR lower urinary tract symptom* OR overactive bladder OR overactive urinary bladder OR Overactive Detrusor)).ti,ab,id. AND ((Infan* OR newborn* OR new-born* OR perinat* OR neonat* OR baby OR baby* OR babies OR toddler* OR minors OR minors* OR boy OR boys OR boyfriend OR boyhood OR girl* OR kid OR kids OR child OR child* OR children* OR schoolchild* OR schoolchild OR school child OR school child* OR adolescen* OR juvenil* OR youth* OR teen* OR under*age* OR pubescen* OR pediatrics OR pediatric* OR paediatric* OR peadiatric* OR school OR school* OR prematur* OR preterm*)).mp. AND ((monitoring/ OR exp behavior modification/OR (Alarm* OR Timed voiding OR Timer* OR bladder sensor* OR bladder notification*)).ti,ab,id. OR ((Urotherap* OR bladder re-education OR bladder rehabilitation OR bladder training OR voiding school* OR voiding class*))).ti,ab,id.

Search terms Cochrane

(urinary incontinence OR urinary incontinen* OR Urinary urge incontinen* OR Daytime incontinen*OR wetting OR Diurnal enuresis OR lower urinary tract symptom* OR overactive bladder OR overactive urinary bladder OR Overactive Detrusor OR urine incontinen* OR bladder incontinen* OR urinary leakage OR urine leakage OR bladder overactivity OR detrusor overactivity OR micturition disorder* OR micturition disturbance* OR lower urinary tract dysfunction AND Infan* OR newborn* OR new-born* OR perinat* OR neonat* OR baby OR baby* OR babies OR toddler* OR minors OR minors* OR boy OR boys OR boyfriend OR boyhood OR girl* OR kid OR kids OR child OR child* OR children* OR schoolchild* OR schoolchild OR school child OR school child* OR adolescen* OR juvenil* OR youth* OR teen* OR under*age* OR pubescen* OR pediatrics OR pediatric* OR paediatric* OR peadiatric* OR school OR school* OR prematur* OR preterm AND Alarm* OR Timed voiding OR Timer*OR bladder sensor* OR bladder notification* OR Urotherap* OR bladder rehabilitation OR bladder training OR voiding school* OR “voiding class” OR “voiding classes”):ti,ab,kw

Search terms Web of Science

"urinary incontinence" OR “urinary incontinen*” OR “Urinary urge incontinen*” OR “Daytime incontinen*” OR “wetting” OR “Diurnal enuresis” OR "Lower Urinary Tract Symptoms" OR “lower urinary tract symptom*”OR “overactive bladder” OR “overactive urinary bladder” OR “Overactive Detrusor” OR “urine incontinen*” OR “bladder incontinen*” OR ”urinary leakage“ OR “urine leakage” OR “bladder overactivity” OR “detrusor overactivity” OR “micturition disorder*” OR ”micturition disturbance*” OR “lower urinary tract dysfunction”

AND

Infan* OR newborn* OR new-born* OR perinat* OR neonat* OR baby OR baby* OR babies OR toddler* OR minors OR minors* OR boy OR boys OR boyfriend OR boyhood OR girl* OR kid OR kids OR child OR child* OR children* OR schoolchild* OR schoolchild OR “school child” OR “school child*” OR adolescen* OR juvenil* OR youth* OR teen* OR under*age* OR pubescen* OR pediatrics OR pediatric* OR paediatric* OR peadiatric* OR school OR school* OR prematur* OR preterm*

AND

Alarm* OR “Timed voiding” OR Timer* OR “bladder sensor*” OR “bladder notification*” OR Urotherap* OR “bladder re-education”OR “bladder rehabilitation “OR “bladder training” OR “voiding school*”OR “voiding class” OR Voiding classes
